# When Words Are Not Enough: Using a QI Framework to Evaluate the Impact of Mentalization-Based Art Psychotherapy for Inpatient Adolescents

**DOI:** 10.1192/bjo.2026.11366

**Published:** 2026-06-30

**Authors:** Catherine Cunning, Tiffany Arnold, Heidi Hales

**Affiliations:** Betsi Cadwaladr University Health Board, Abergele, United Kingdom

## Abstract

**Aims:**

To establish a weekly Mentalization Based Art Psychotherapy Group on Kestrel Ward, North Wales Adolescent Service.

To achieve 75% attendance from young people admitted to the ward between May and September 2025.

**Methods:**

We decided to use the Quality Improvement ‘Plan, Do, Study, Act’(PDSA) cycle to assist in evaluating the process of setting up and maintaining a sustainable and well-functioning Mentalization Based Art Psychotherapy Group.

We completed 4 PDSA cycles looking at establishing the group, engaging difficult to reach young people and promoting staff understanding of the group.

**Results:**

A weekly inpatient Mentalization-based art psychotherapy group was established, with 13 group sessions taking place over a period of 17 weeks. During this process facilitators reflected on how to adapt the group to both the setting and the patient cohort. There is no clear blueprint for this type of intervention within the inpatient setting and to establish the group it was necessary to innovate. This meant working in an adaptive manner based on the needs and opinions of the young people present on the ward at any given time.

Working the patients' way was clearly effective, but it was challenging to do so in a stressful and busy ward environment that was not always conducive to staff or patient's mentalizing processes.

The group has now become well established and sustainable with most young people who are admitted to the ward attending on a weekly basis. It is hoped that engagement will be further improved through continuity and gaining increased input from young people with regards to liked activities.

**Conclusion:**

To ensure the on-going provision of the Mentalization Based Art Psychotherapy Group the facilitators recommend the following:

Group provision is continued on a weekly basis.

The availability of a practitioner with a background in psychological therapies/psychiatry is essential to co-facilitate alongside the art therapist (and this must consistently be the same person).

Dedicated specialist clinical supervision for group facilitators is provided monthly.

Active planning takes place in relation to provision of a suitable therapeutic group space and facilities.

There is provision of staff training and supervision based on mentalizing principles.

